# Specific Gene *bciD* for C7-Methyl Oxidation in Bacteriochlorophyll *e* Biosynthesis of Brown-Colored Green Sulfur Bacteria

**DOI:** 10.1371/journal.pone.0060026

**Published:** 2013-04-01

**Authors:** Jiro Harada, Tadashi Mizoguchi, Souichirou Satoh, Yusuke Tsukatani, Makio Yokono, Masato Noguchi, Ayumi Tanaka, Hitoshi Tamiaki

**Affiliations:** 1 Department of Medical Biochemistry, Kurume University School of Medicine, Kurume, Fukuoka, Japan; 2 Graduate School of Life Sciences, Ritsumeikan University, Kusatsu, Shiga, Japan; 3 Institute of Low Temperature Science, Hokkaido University, Sapporo, Hokkaido, Japan; 4 Precursory Research for Embryonic Science and Technology, Japan Science and Technology Agency, Kawaguchi, Saitama, Japan; 5 Core Research for Evolutional Science and Technology, Japan Science and Technology Agency, Sapporo, Hokkaido, Japan; US Naval Reseach Laboratory, United States of America

## Abstract

The gene named *bciD*, which encodes the enzyme involved in C7-formylation in bacteriochlorophyll *e* biosynthesis, was found and investigated by insertional inactivation in the brown-colored green sulfur bacterium *Chlorobaculum limnaeum* (previously called *Chlorobium phaeobacteroides*). The *bciD* mutant cells were green in color, and accumulated bacteriochlorophyll *c* homologs bearing the 7-methyl group, compared to C7-formylated BChl *e* homologs in the wild type. BChl-*c* homolog compositions in the mutant were further different from those in *Chlorobaculum tepidum* which originally produced BChl *c*: (3^1^ *S*)-8-isobutyl-12-ethyl-BChl *c* was unusually predominant.

## Introduction

Chlorophyll(Chl)s and bacteriochlorophyll(BChl)s are key pigments for the initial stage of photosynthetic processes, harvesting sunlight, transferring excited energy, and performing charge separation. Each (B)Chl pigment has a distinctive absorption character in ultraviolet, visible, and near-infrared regions which is dependent on the molecular structure [1]. Photosynthetic organisms live under various light conditions and use one or two (B)Chls species for efficient absorption of habitat- or niche-specific light. All oxygenic photosynthetic organisms commonly possess Chl *a* (or 8-vinyl-Chl *a* in the case of *Prochlorococcus*). In addition to Chl *a*, plants and green algae have Chl *b*, and some cyanobacteria possess Chl *d* [2] or Chl *f* [3]. Chls *b*, *d*, and *f* have different absorption properties from Chl *a* (Q_y_ and Soret maxima = 660.8 and 429.6 nm in diethyl ether) due to the presence of a formyl group at the C7, C3, and C2 positions of chlorin ring, respectively (Q_y_ and Soret maxima for; Chl *b*, 642.2/453.0 nm; Chl *d*, 685.8/445.8 nm; Chl *f*, 694.5/439.5 nm [4]). Among Chls *b*, *d*, and *f*, a gene encoding enzyme for formylation at C7 of Chl *b* has already been identified as chlorophyllide (Chlide) *a* oxygenase gene, *CAO* [5]. It was observed that the mutation of *CAO* gene in the green algae *Chlamydomonas reinhardtii* synthesized no Chl *b*, but exclusively Chl *a*, and a recombinant CAO enzyme converted the methyl group at C7 of Chlide *a* to the 7-formyl group of Chlide *b* via a hydroxymethyl group by two successive monooxygenations *in vitro* [6]. In contrast, the genes for formylation in Chls *d* and *f* have not yet been found. Elucidation of the genes encoding the oxidation to a formyl group is significant to understand the evolution of (B)Chl pigment biosynthesis as well as the photosynthetic mechanism.

**Figure 1:**
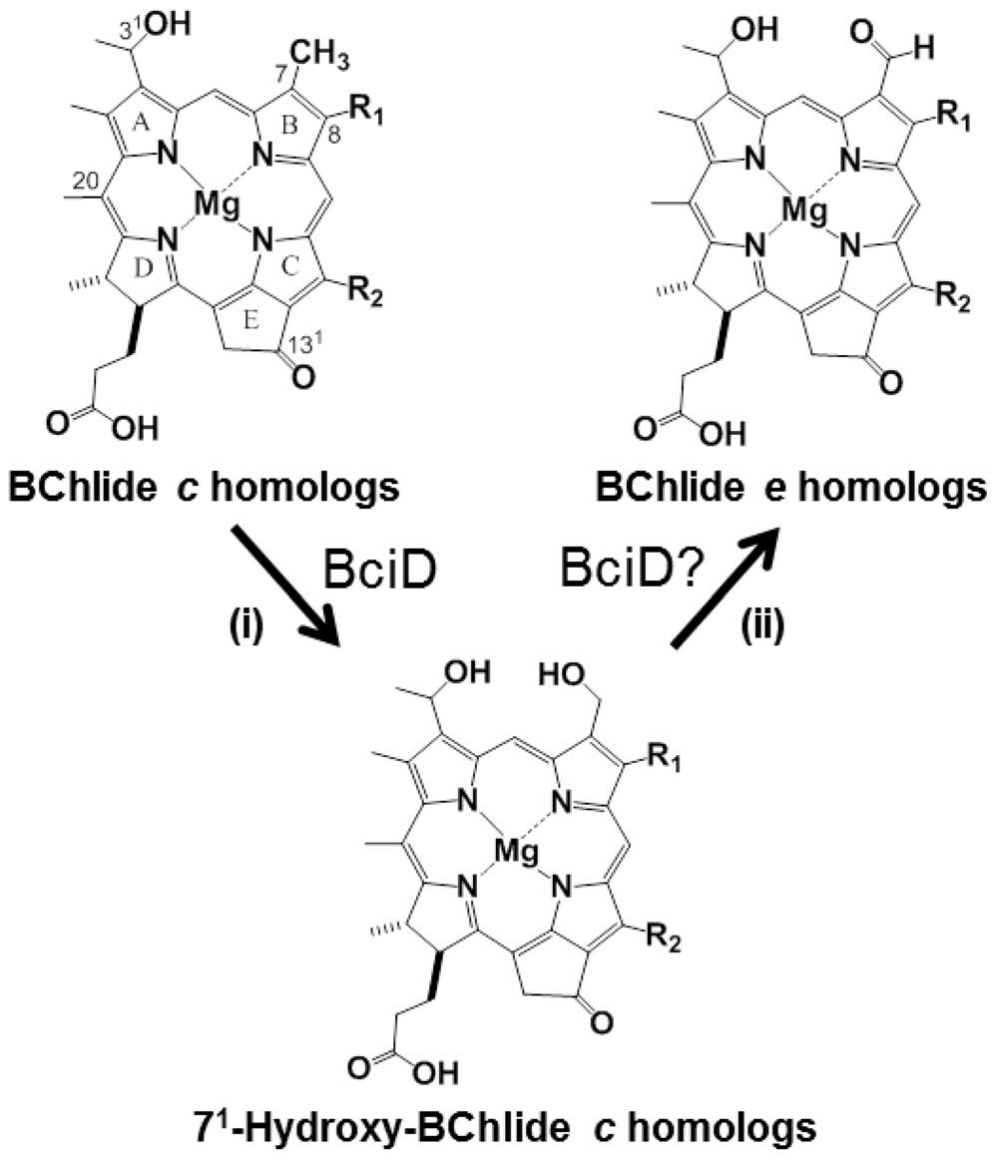
Proposed oxidization of a methyl group at the C7 position of BChlide *c* to a formyl group in BChl *e* biosynthesis: (i) monooxygenation of the 7-methyl to 7-hydroxymethyl group; (ii) monooxygenation of the 7-hydroxymethyl to 7-dihydroxymethyl group followed by the spontaneous dehydration to the 7-formyl group or direct dehydrogenation of the 7-hydroxymethyl to 7-formyl group. The C3^1^-stereochemistry is *R* or *S*; R^1^ =  ethyl, propyl, or isobutyl; R^2^ =  methyl or ethyl.

Among BChl molecules found in anoxygenic photosynthetic bacteria in natural environments, BChl *e* is the only formylated pigment at the C7 position (see Fig. 1). BChls *c* and *d* as well as BChl *e* are found in green sulfur bacteria (GSB) [1], [7]. These pigments self-aggregate to form characteristic extra-membrane antenna systems, called chlorosomes. In a chlorosome, a large amount of BChl *c*, *d*, or *e* molecules give highly-ordered and protein-free suprastructures, and permit efficient absorption of light and rapid migration of excitation energy. The biosynthesis of BChls *c* and *d* has been investigated using *Chlorobaculum* (*Cba.*) *tepidum*, a genetically tractable model organism of GSB [8], [9]. Following the recent discovery of an enzyme for removal of the 13^2^-methoxycarbonyl group from Chlide *a*, called BciC [10], all the genes encoding BChls *c* and *d* biosynthetic enzymes were revealed. However, only one step in the biosynthesis of BChl *e* has been unidentified, i.e., an enzyme producing a formyl group at the C7 position of bacteriochlorophyllide (BChlide) *e* (Fig. 1) was not found, as was also true in the cases of Chls *d* and *f* mentioned above.

Very recently, the mutagenesis of one species of the GSB *Cba. limnaeum* [phylogenetically renamed from *Chlorobium* (*Chl.*) *phaeobacteroides* [11]] which synthesized BChl *e*, was achieved based on a double cross-over event between homologous regions by natural transformation [12], [13], or a single cross-over event by the *E. coli-*conjugation system [14]. In these studies, a mutant deleting C20-methyltransferase gene (*bchU*) for BChl *e* synthesis was constructed, and analysis of its pigment compositions showed accumulation of C20-unsubstituted BChl *f*, whose name and structure were proposed, based on BChl *e* which was discovered in 1975 [15], but the BChl *f* has never been observed in nature [16]. In the mutant cells, BChl *f* self-aggregates were formed chlorosomes and could transfer their harvested light energy to BChl *a* associated with baseplate proteins as an initial energy acceptor in a chlorosome. Therefore, BChl *f* was assigned as a photosynthetically active pigment in the bacterial cells. The mutagenesis of *Cba. limnaeum* opens the door for the discovery of genes for BChl *e* biosynthesis.

In this study, we describe the identification of a gene denoted *bciD* which encodes an enzyme requisite for the C7-methyl oxidation (formylation) in BChl *e* synthesis. The *bciD* gene was initially identified by performing phylogenetic profiling analysis of genomic DNA sequence data among BChl *e* producing strains in GSB. To verify that BciD played a role in BChl *e* biosynthesis, a null mutant of *Cba. limnaeum* was constructed by deleting the *bciD* gene. This mutant was unable to synthesize BChl *e*, instead accumulating BChl *c*. By characterizing the BChl compositions, we show that BciD is essential for the oxidation to a 7-formyl group in BChl *e* biosynthesis, and discuss the enzymatic function of BciD and its role in biosynthetic pathway. This work was preliminarily reported by our group [17], [18], and independently, a similar result was appeared in the oral presentation by Prof. Bryant [19].

## Materials and Methods

### Bacterial Strains and Culture Conditions

The strain RK-j-1 [13] of *Cba. limnaeum* was used as a parent wild type strain. *Cba. tepidum* WT2321 [20] was used as a control having BChl *c* homologs for HPLC analyses. These bacteria were anaerobically grown in a 30 mL or 1 L screw-capped bottle with liquid CL medium, or on a solid CP plate [21]. The growth temperature was adjusted to 30°C for *Cba*. *limnaeum* or 45°C for *Cba. tepidum*. *E. coli* DH5α was grown in LB medium containing 100 µg/mL of ampicillin or 20 µg/mL of streptomycin.

### Plasmid Constructions

**Figure 2:**
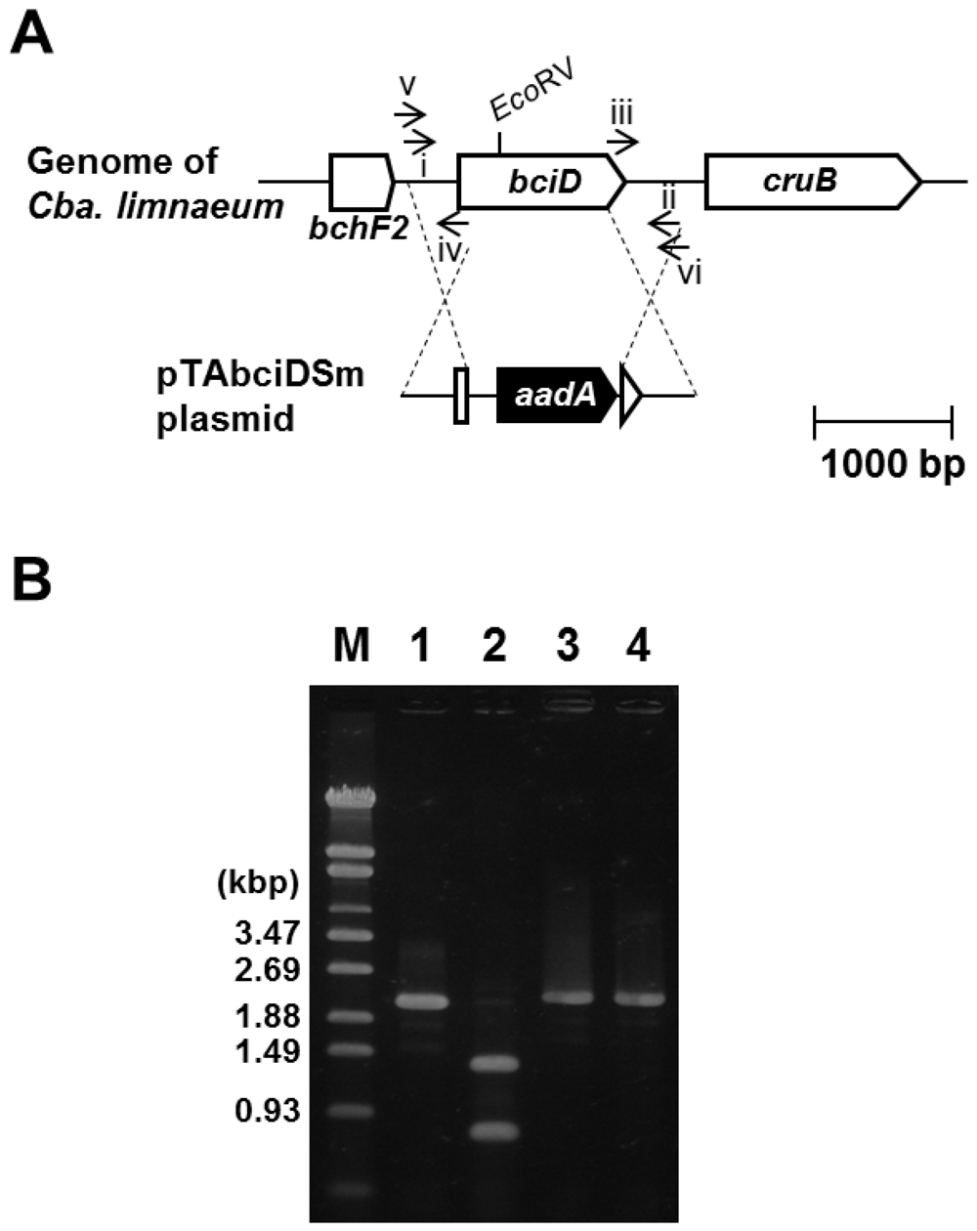
Construction of *Cba. limnaeum bciD* gene inactivated mutant. (A) Schematic map of genes arrangement around *bciD* gene in the genome of *Cba. limnaeum* RK-j-1, and its insertional inactivation. The *aadA1* gene, conferring resistance to streptomycin and spectinomycin, was inserted in *bciD*. Arrows represent the primers bciD-F (i), bciD-R (ii), bciD-inf-F (iii), bciD-inf-R (iv), bciD-comf-F (v), and bciD-comf-R (vi). (B) PCR confirmation of gene interruption. The *bciD* gene was amplified from genomic DNA extracted from the wild type (lanes 1 and 2) and a mutant (lanes 3 and 4) of *Cba. limnaeum*, using above bciD-comf-F and -R primers. The products in lanes 2 and 4 were then digested by restriction enzyme *Eco*RV, and the fragments yielded from wild type and the mutant were 1.36 and 0.78, and 2.22 kbp, respectively. Lane M, molecular size marker (the sizes of bands are indicated at left).

The draft genome sequence of *Cba. limnaeum* strain RK-j-1 has been determined and will be reported elsewhere (J. Harada et al., in preparation). Complete DNA sequences of the *bciD* gene and its flanking genes were deposited in the GenBank (Accession No. AB762294). To construct the *Cba. limnaeum* mutant lacking BciD (Fig. 2A), pTAbciDSm plasmid was produced as follows. The *aadA* gene, conferring resistance to streptomycin and spectinomycin, was amplified from the plasmid pHP45Ω [22] by PCR using the primer set aadA-F (CTGTTCGGTTCGTAAGCTGT) and aadA-R (CGTCGGCTTGAACGAATTGT). While, a 1.99-kbp DNA fragment containing the *bciD* gene was amplified from the genomic DNA of *Cba. limnaeum* by PCR using bciD-F (TCACTGTTATGTTGTCGGGTA) and bciD-R (GGTAAGCACCTATGCCGAAA) primers. The PCR product containing *bciD* was cloned into the TA-cloning site of T-Vector pTA2 (TOYOBO, Japan), yielding pTA2-bciD plasmid. To amplify the DNA fragment from pTA2-bciD without the inner portion of *bciD*, the plasmid was used as the template for PCR with primers bciD-inf-F (TCGTTCAAGCCGACG TCTTGCCAAGGATCATCGTC) and bciD-inf-R (TTACGAACCGAACAG TTGTTAACCGTCACCTTGGC). Underlined sequences of these primers are designed to overlap with partial DNA sequences of the above-mentioned aadA-F and aadA-R primers for the following In-Fusion cloning. The resulting PCR product and the PCR fragment containing the *aadA* gene were ligated using an In-Fusion® HD Cloning Kit (Clontech, USA), creating pTAbciDSm.

### Transformation of *Cba. limnaeum*

Natural transformation using the *Cba. limnaeum* RK-j-1 strain was performed as previously described [13]. About 0.1 mg of *Eco*RI-digested pTAbciDSm was used for the transformation. CP plates, containing both 100 µg/mL of streptomycin and 150 µg/mL of spectinomycin, were used for selection of transformants.

PCR analysis was carried out to monitor segregation of wild type and mutant alleles using bciD-comf-F (CATCATAGGGGGGCAATAGA) and -R (CTTGCCCGGAGAAGGATTAT) primers. A DNA molecular weight marker, λ/*Sty*I digest (TAKARA, Japan), was used for molecular mass estimations of PCR products. DNA sequence analyses of the PCR fragments were determined using an ABI PRISM® 3100 Genetic Analyzer (Applied Biosystems, USA).

### Determination of Compositions of BChl *c* Homologs

Pigments of the *Cba. limnaeum* mutant strain were extracted and analyzed as follows. A mixture of acetone and methanol (9∶1, v/v) was added to the harvested cells and mixed using a vibrator. A mixture of diethyl ether and petroleum ether (1∶1, v/v) and then distilled water were added to transfer the pigment components to the ether layer. The ether phase was collected and evaporated to dryness under a stream of N_2_ gas, and the residues were dissolved in a small amount of high performance liquid chromatography (HPLC) eluent, described below. Liquid chromatography mass spectrometry (LCMS) analysis was performed using a Shimadzu LCMS-2010EV system (Shimadzu, Japan) comprising a liquid chromatograph (SCL-10Avp system controller, LC-10ADvp pump, and SPD-M10Avp photodiode-array detector) and a quadrupole mass spectrometer equipped with an atmospheric pressure chemical ionization (APCI) probe. Reverse-phase HPLC was performed under the following conditions: column, Cosmosil 5C_18_-AR-II (4.6 φ×150 mm, Nacalai Tesque, Japan); eluent, acetonitrile:acetone: H_2_O (65∶15:20, v/v/v); flow rate, 1.0 mL/min; detection wavelength, 415, 435 and 465 nm. APCI-MS spectra were measured as follows: resolution, ±0.15 Da; capillary temperature, 250°C; APCI vaporizer temperature, 400°C; ionization voltage, 4.5 kV; sheath gas flow, 2.5 L/min; drying gas pressure, 0.02 MPa.

## Results

### Identification of Candidate Gene for C7-Methyl Oxidation in BChl *e* Biosynthesis

To identify candidate genes encoding enzymes specifically involved in BChl *e* biosynthesis, we first performed *Chlorobi*-specific BLAST analysis, a program equipped by the Joint Genome Institute (JGI: http://genome.jgi.doe.gov/pages/blast.jsf?db=chlorobi), using the *CAO* gene of *Chlamydomonas reinhardtii* as a query [5]. The results showed no hit of a similar gene of *CAO* among genomes of eleven GSB spices containing three brown-colored BChl *e*-producing strains, *Chl. phaeobacteroides* BS1, *Chl. phaeobacteroides* DSM266, and *Pelodictyon phaeoclathrathiforme* BU-1.

Then, we used a computer-aided gene discovery program, Correlation Coefficient Calculation Tool (CCCT) [23], to find candidate genes for C7-methyl oxidase in BChl *e* biosynthesis. CCCT is based on a comparative analysis of whole gene sets between different organisms, which allows for the identification of genes involved in a particular function in a certain class of organisms [23], [24]. In this study, we made two assumptions: (1) the above three strains producing BChl *e* must have a specific enzyme(s) responsible for BChl *e* synthesis; (2) the enzyme(s) would not be found in any other GSB that possesses BChl *c* or *d* and filamentous anoxygenic phototrophic bacteria containing BChl *c*. The program was used with gene sets from 9 species whose genome sequences have been determined (see Table S1). Here, ORFs of *Chl. phaeobacteroides* BS1 were used as query sequences. In the output data, all ORFs of strain BS1 were ranked according to their correlation coefficients, such that ORFs specifically conserved in the three strains of BChl *e*-producing bacteria were expected to show higher values. In the top genes of the output data (Table S2), Cphamn1_0270 in BS1 strain, Ppha_2747 in BU-1 strain and Cpha266_0196 in DSM266 strain had a higher similarity with one another, and were annotated as radical *S*-adenosyl-L-methionine (SAM) enzymes. The gene was found only in the three brown-colored bacteria but not in the other 6 bacteria. The conversion from a methyl to formyl group at the C7 position is suggested to involve in radical reactions [25]. Further, the radical SAM gene in each genome of these strains is interestingly present in the very similar gene cluster containing the brown-colored GSB specific genes for cyclization of γ-carotene, *cruB* [26], and C3-vinyl hydratase(BchF)-like gene (here called *bchF2*) (see Fig. 2A). Therefore, we chose this radical SAM gene as a good candidate for a gene coding C7 formylation (methyl oxidation) enzyme. This proposal was supported by another bioinformatic analysis [27]. The radical SAM gene was designated *bciD*.

### Construction of bciD Mutant of Cba. limnaeum RK-j-1

We next tested whether *bciD* encodes the enzyme oxidizing the 7-methyl to formyl group involved in BChl *e* biosynthesis. Mutational analysis is effective in investigating the function of an unknown gene, but the above three BChl *e*-producing strains could not be utilized for such genetic studies. Therefore, we used the brown-colored BChl *e*-producing GSB *Cba. limnaeum* RK-j-1 whose mutagenesis method by natural transformation was recently established [13]. For this study, we determined the genomic sequence of *Cba. limnaeum* RK-j-1 strain, and performed a BLAST search analysis using BciD amino acid sequence as the query. The result showed that the gene cluster conserved among the above three strains as well as the *bciD* gene, was also observed in the genome of the RK-j-1 strain. Thus, the *bciD* gene of this strain was inactivated by insertion of the *aadA* streptomycin and spectinomycin resistance cassette, as shown in Fig. 2A. To confirm the mutation, PCR analysis was performed using primers bciD-comf-F and -R, and then an amplified DNA fragment containing *bciD* gene and its flanking genes was digested with a restriction enzyme *Eco*RV. Agarose gel electrophoresis analysis (Fig. 2B) showed *Eco*RV-treated 1.36- and 0.78-kbp DNA fragment bands of wild type (lane 2), and while non-digested 2.22-kbp DNA band of the *bciD* mutant (lane 4), because a large part of the *bciD* gene containing *Eco*RV site was replaced with the *aadA* cassette. This result indicated that the *aadA* gene was correctly introduced into the *bciD* gene in the mutant strain. We also confirmed this mutation by DNA sequence analysis of the PCR products. These results indicated that the *bciD* mutant was completely segregated.

### Absorption Properties of *bciD* Mutant of *Cba. limnaeum*

**Figure 3:**
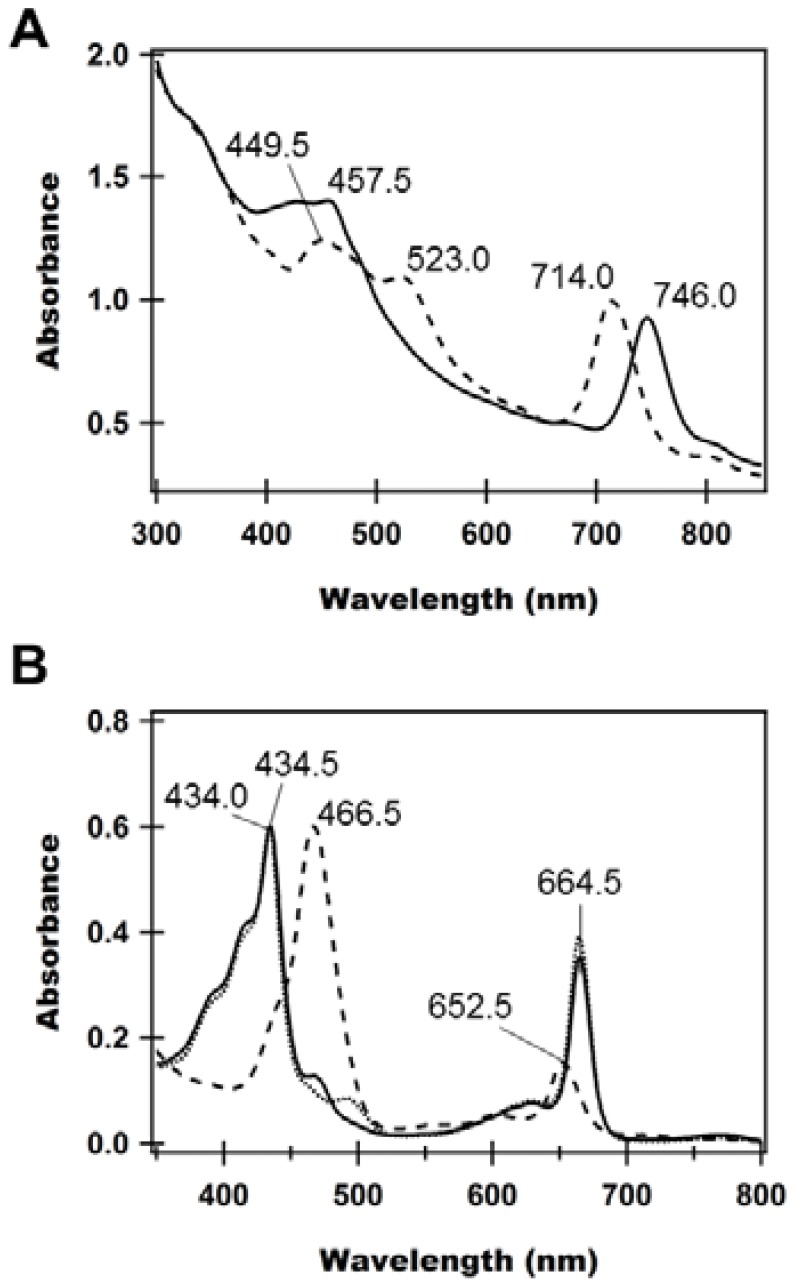
UV-Vis-NIR absorption spectra of whole cells (A) and extracted pigments (B) of the *Cba. limnaeum* wild type (broken lines) and *bciD* mutant (solid lines); measured using a Hitachi UV-2550 (Shimadzu, Japan) spectrophotometer. (A) Wild type and mutant cells in stationary phase were collected and suspended in 50 mM Tris-HCl (pH 7.8) containing 150 mM NaCl; normalized at 660 nm. (B) Pigments were extracted with a mixture of acetone and methanol (7∶2, v/v), and used for the measurements; normalized at Soret maxima. The dotted line shows absorption spectrum of pigments from *Cba. tepidum* as control for BChl *c*.

Unlike the brown-color wild type cells of *Cba. limnaeum*, the *bciD* mutant cells showed green-color similar to GSB species containing BChl *c* or *d* (Figure S1). Figure 3A shows the UV-Vis-NIR absorption spectra of whole cells of the wild type (broken line) and the mutant (solid line). The Q_y_ and Soret bands of the mutant were observed at 746.0 and 457.5 nm, respectively. These absorption bands displayed maxima different to those the wild type, especially, the Q_y_ peak of the *bciD* mutant, red shifted by 32 nm relative to the wild type. These changes in absorption spectra by the mutation were reminiscent of GSB producing BChl *c* (Q_y_ peak = 745–755 nm) [24], [28]. Figure 3B shows the absorption spectra of pigments extracted from the wild type (broken line) and *bciD* mutant (solid line) of *Cba. limnaeum*, together with the spectrum from *Cba. tepidum* cells (dotted line) as control for BChl *c* in a mixture of acetone and methanol. The spectrum of the mutant was quite different from that of the wild type containing BChl *e*, but was identical to *Cba. tepidum* containing BChl *c* with the exception of the carotenoid region (∼450–500 nm). These results strongly indicated that the *bciD* mutant cells accumulated BChl *c* as its dominant composite pigment.

### Determination of Compositions of BChl c Homologs in Cba. limnaeum bciD Mutant

**Figure 4:**
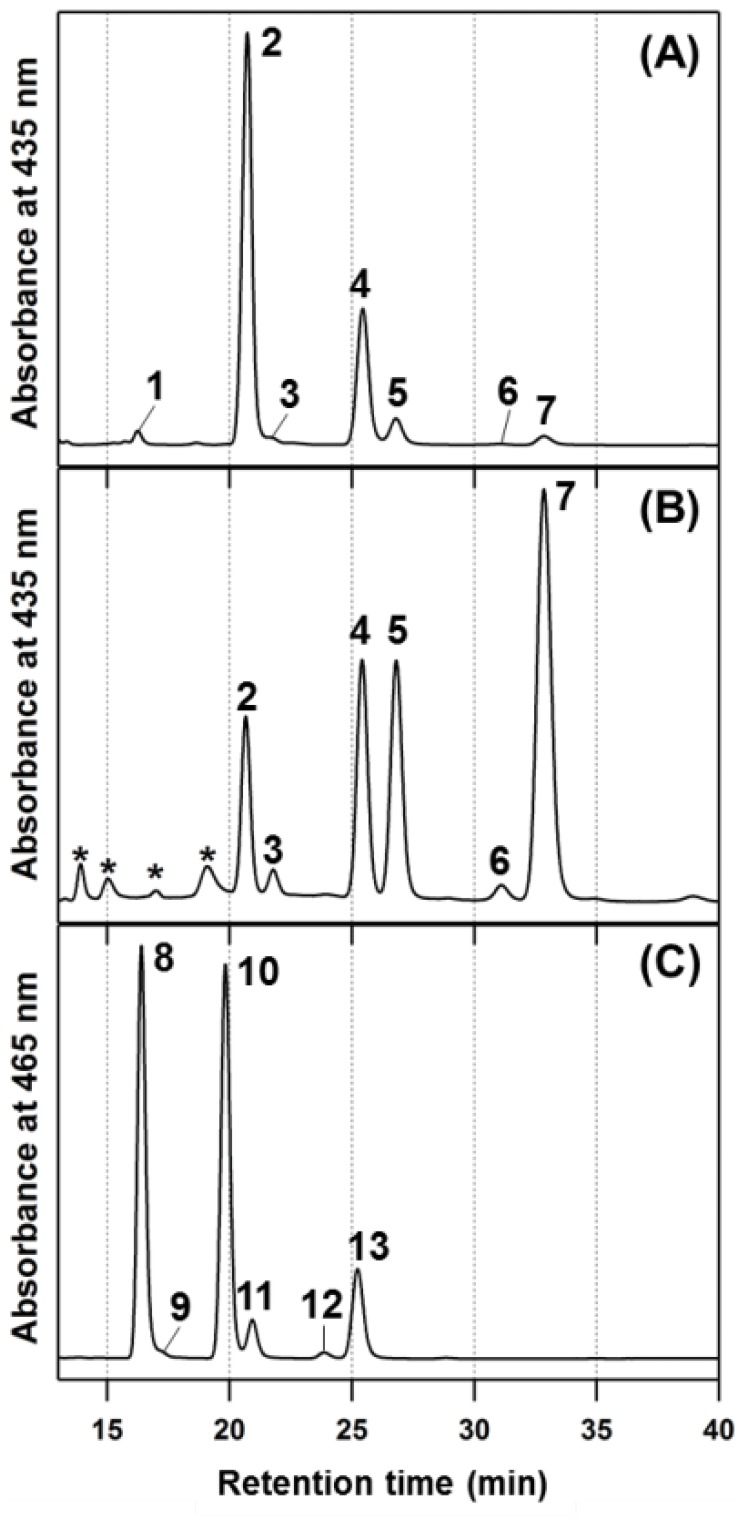
HPLC elution profiles of extracted pigments from GSB. (A) From *Cba. tepidum* as control for BChl *c*, recorded at 435 nm. (B) From *Cba. limnaeum bciD* mutant recorded at 435 nm. (C) From *Cba. limnaeum* wild type recorded at 465 nm. Peak 1, R[E,M]BChl *c*; peak 2, R[E,E]BChl *c*; peak 3, S[E,E]BChl *c*; peak 4, R[P,E]BChl *c*; peak 5, S[P,E]BChl *c*; peak 6, R[I,E]BChl *c*; peak 7, S[I,E]BChl *c*; peak 8, R[E,E]BChl *e*; peak 9, S[E,E]BChl *e*; peak 10, R[P,E]BChl *e*; peak 11, S[P,E]BChl *e*; peak 12, R[I,E]BChl *e*; peak 13, S[I,E]BChl *e*. Peaks at the asterisk in panel (B) indicate impurities produced during handling of the sample.

Pigments extracted from the wild type and mutant cells of *Cba. limnaeum*, as well as *Cba. tepidum* cells were analyzed by APCI-LCMS (Fig. 4 and Table 1). The elution profiles of the pigments from *Cba. tepidum* contained seven types of BChl *c* (see Fig. 4A) possessing an *R* or *S* stereo-configuration at the C3^1^-asymmetric position, and different degrees of methylation at the C8^2^ and C12^1^ positions: (3^1^ *R*)-8-ethyl-12-methyl-(R[E,M])BChl *c* (peak 1), (3^1^ *R*)-8-ethyl-12-ethyl-(R[E,E])BChl *c* (peak 2), (3^1^ *S*)-8-ethyl-12-ethyl-(S[E,E])BChl *c* (peak 3), (3^1^ *R*)-8-propyl-12-ethyl-(R[P,E])BChl *c* (peak 4), (3^1^ *S*)-8-propyl-12-ethyl-(S[P,E])BChl *c* (peak 5), (3^1^ *R*)-8-isobutyl-12-ethyl-(R[I,E])BChl *c* (peak 6), and (3^1^ *S*)-8-isobutyl-12-ethyl-(S[I,E])BChl *c* (peak 7). In HPLC profiles of the *bciD* mutant cells of RK-j-1, six peaks, 2 to 7, corresponding to the above BChl *c* in *Cba. tepidum* were detected as R[E,E]BChl *c*, S[E,E]BChl *c*, R[P,E]BChl *c*, S[P,E]BChl *c*, R[I,E]BChl *c*, and S[I,E]BChl *c* (Fig. 4B). These six components exhibited the same retention times, mass numbers, and fragmentation patterns in APCI-LCMS as those of the authentic BChls *c* in *Cba. tepidum* (summarized in Table S3), indicating that the *bciD* mutant accumulated BChl *c* homologs. However, the compositional pattern of BChl *c* homologs in the mutant cells were greatly different from that in *Cba. tepidum*: the former synthesized a significant amount of C3^1^ *S* epimers and more highly C8^2^-methylated BChl *c* pigments than the latter. The *bciD* mutant cells contained S[I,E]BChl *c* (peak 7) as a major BChl species and also showed comparable amounts of two epimers, R- and S[P,E]BChls *c* (peaks 4 and 5); in contrast, *Cba. tepidum* cells synthesized R[E,E]BChl *c* (peak 2) as its main pigment and a trace amount of S[I,E] homolog.

**Table 1:** Compositions of BChl species in *Cba. limnaeum* wild type and *bciD* mutant, and *Cba. tepidum*.

Strains	BChl (%)							Total (%)	
	R[E,M]	R[E,E]	S[E,E]	R[P,E]	S[P,E]	R[I,E]	S[I,E]	3^1^ *R*/*S* a	8-E/P/I^b^
*Cba. tepidum* wild type (BChl *c*)	1.8	64.8	0.1	25.6	5.5	0.1	2.1	92.3/7.7	66.7/31.1/2.2
*Cba. limnaeum bciD* mutant (BChl *c*)	0.0	12.6	1.9	19.6	20.9	1.2	43.8	33.4/66.6	14.5/40.5/45.0
*Cba. limnaeum* wild type (BChl *e*)	0.0	36.1	4.6	39.9	5.3	1.1	13.0	77.1/22.9	40.7/45.2/14.1

a Ratio of 3^1^ *R* and 3^1^ *S* configurations of BChl species.

b Ratio of ethyl (E), propyl (P), and isobutyl groups (I) at C8 of BChl species.

The *bciD* mutant cells gave no detectable amount of BChl *e* homologs, R/S[E,E]-, R/S[P,E]-, R/S[I,E]BChl *e*, that were seen in the wild type of *Cba. limnaeum* as peaks 8/9, 10/11, and 12/13, respectively (Fig. 4C). The pigment analysis indicated that the *bciD* gene was responsible for the C7 formylation (methyl oxidation) in the BChl *e* biosynthetic pathway.

## Discussion

The *bciD* gene-inactivated mutant of *Cba. limnaeum* was constructed and its pigments were analyzed. This mutant cells did not synthesize BChl *e* pigments, but accumulated BChl *c* species which were found in *Cba. tepidum* cells producing BChl *c* homologs, although the composition of BChl *c* homologs was largely different between both cells. The ratio of C3^1^ *R* and *S* epimers of BChl species in this mutant was 33/67 (*S*-rich), while those in *Cba. tepidum* cells showed *R*-rich 92/8 as well as in *Cba. limnaeum* wild type, 77/23 (Table 1). The homolog composition of ethyl, propyl, and isobutyl groups at the C8 position in *bciD* mutant showed “14/41/45” and was different from its wild type “41/45/14”. Since the epimer and homolog compositions of the C20-unsubstituted BChl *f* pigments in the *bchU* mutant of *Cba. limnaeum* were almost the same as those of BChl *e* in the wild type cells [13], the present formation of *S*-rich epimers and isobutyl-rich homologs were ascribable to the alteration of the C7 substituent (7-methyl to formyl group) by the mutation.

It was reported that C3-vinyl hydrases, BchF and BchV converted the vinyl group at C3 position to the 1-hydroxyethyl group with *R* and *S* configurations, respectively [29]. The enzymatic activity of the BchV in *Cba. limnaeum* over the BchF might increase for the hydration of a C7-methyl substrate, in comparison with that of a C7-formyl derivative. Methylation at the C8^2^ position was catalyzed by a methylase BchQ to afford propyl and isobutyl groups from the 8-ethyl group [30]. The BchQ enzyme would have a higher activity against C7-methyl substrates than C7-formyl ones. Moreover, the alteration of pigment compositions of the *bciD* mutant should be attributable to the activity of BChl synthase for BChl *e*. It is known that BChl *c* synthase (BchK) [31], as well as BChl *a* and Chl synthases (BchG and ChlG, respectively), esterified the C17-propionate of (B)Chlides with a long aliphatic chain and recognized a cyclic tetrapyrrole structure of each (B)Chl species, showing a strict substrate specificity [32]. In *Cba. limnaeum* cells, the BChl *e* synthase would recognize the 7-formyl group of BChlide *e*, while in the case of *bciD* mutant, this enzyme seemed to catalyze farnesyl-esterification of S[I,E]BChlide *c* possessing a bulky substituent and lacking an electron-withdrawing group at the B ring more favorably than any other species.The mutational study indicates that BciD is involved in the oxidation of the 7-methyl group in BChl *e* biosynthesis, but the reaction mechanism of this enzyme is unclear at present. Based on the CAO-catalyzed oxidation in the biosynthesis from Chl *a* to *b* [6], it is considered that the 7-methyl group of BChlide *c* is converted to the 7-formyl group of BChlide *e* by two-step monooxygenation (Fig. 1). The accumulation of BChl *c* species in the *bciD* mutant shows that BciD is necessary for the first-step of oxygenation [(i) in Fig. 1], but it is unclear whether BciD catalyzes the second-step of oxidase reaction [(ii) in Fig. 1]. No detection of 7^1^-hydroxy-BChl *c* in the present LCMS also supports the participation of BciD in the first oxidation. The CAO-mediated two-step reactions required radical species and oxygen molecule [25]. The BciD protein belongs to the radical SAM family and can initiate radical reactions. Since GSB including *Cba. limnaeum* are strictly anaerobes, water molecule will be used for a BciD-mediated reaction as an oxygen source. In BChl biosynthesis, a BchE enzyme catalyzes to add an oxo-group at the C13^1^ position using water under anaerobic growth conditions, and successively forming the E ring of protochlorophyllide (see Fig. 1) [33], [34]. To clarify the enzymatic properties of BciD, further molecular genetics and biochemical studies including its expression in BChl *c*-producing GSB cells and *in vitro* oxidation by purified BciD protein are required.

The BciD enzymes were highly conserved among BChl *e*-possessing GSB (identity >86%, similarity >93%, using *Cba. limnaeum* BciD as query) as mentioned above. We found the paralogs of *bciD* in various organisms by performing a BLAST analysis using the *bciD* of *Cba. limnaeum* as query. The paralogs belonged to the radical SAM family and were distributed in photosynthetic (Fig. S2) and non-photosynthetic bacteria, but the identities of overall proteins were low (<30%). Such *bciD* paralogs were found in genomes of some purple bacteria including *Rhodopseudomonas* (*Rps.*) *palustris* (Rpal_2885 of TIE-1 strain, identity/similarity = 26/45%), and *Rhodobacter* (*Rba.*) species (RCAP_rcc02237 of *Rba. capsulatus* SB1003, 24/44%). In cyanobacteria, the paralogs of *bciD* were also present: *Synechococcus* sp. PCC7335 (S7335_2575, 30/48%), *Synechocystis* sp. PCC6803 substrain PCC-N (s110785, 26/48%), and Chl *d*-producing *Acaryochloris marina* MBIC11017 (AM1_2229, 24/46%). It should be reconfirmed that no *bciD* paralogs were seen in genomes of GSB and filamentous anoxygenic phototrophic bacteria producing BChls *c* and/or *d*. Functional analyses for these paralogs will be useful to understand the evolutions of BciD involved in (B)Chl pigment biosynthesis as well as radical SAM enzymes.

## Supporting Information

Figure S1 **A photograph of liquid cultures of wild type (left) and** ***bciD*** **mutant (right) grown under phototrophic conditions.**
(DOC)

Figure S2 **Phylogenetic analysis of** ***bciD*** **paralogs among photosynthetic bacteria.** A neighbor-joining tree was constructed with translated sequences of *bciD* paralogs that showed over 1e-10 of the BLASTP e-value. Bootstrap values for each clade were obtained by 1500 replications, and indicated. The accession numbers of sequences to construct the tree are as follows: *Acaryochloris marina* MBIC11017, YP_001516556; *Acaryochloris* sp. CCMEE 5410, ZP_09251378; *Chl. phaeobacteroides* BS1, YP_001958720; *Chl. phaeobacteroides* DSM266, YP_910687; *Pelodictyon phaeoclathratiforme* BU-1, YP_002019518; *Prochlorococcus marinus* MIT9301, YP_001091538; *Rba. capsulatus* SB 1003, YP_003578374; *Rhodomicrobium vannielii* ATCC17100, YP_004013831; *Rba. sphaeroides* ATCC17025, YP_001166986; *Rps. palustris* CGA009, NP_947956; *Rps. palustris* TIE-1, YP_001991868; *Rps. palustris* BisA53, YP_782106; *Rubrivivax gelatinosus* IL144, YP_005437386; *Synechococcus elongatus* PCC6301, YP_171412; *Synechococcus elongatus* PCC7942, YP_399856; *Synechococcus* sp. CC9605, YP_381421; *Synechococcus* sp. PCC7002, YP_001733926; *Synechococcus* sp. PCC7335, ZP_05036141; *Synechococcus* sp. WH5701, ZP_01084318; *Synechococcus* sp. WH 7805, ZP_01123862; *Synechocystis* sp. PCC6803, NP_442645.
(DOC)

Table S1 **GSB whose genome sequences were used for CCCT analysis.**
(DOC)

Table S2 **Top 5 genes specifically conserved among brown-colored GSB, that was calculated by CCCT.**
(DOC)

Table S3 **APCI-mass spectrometric data of BChl** ***c*** **and** ***e*** **homologs found in the full-growth cells of the wild type and** ***bchU*** **mutant of** ***Cba. limnaeum*** **.**
(DOC)
