# Supplementary material for: Specific Gene bciD for C7-Methyl Oxidation in Bacteriochlorophyll e Biosynthesis of Brown-Colored Green Sulfur Bacteria
Source: PLoS One. 2013 Apr 1;8(4):e60026. doi: 10.1371/journal.pone.0060026 (PMC3613366; doi:10.1371/journal.pone.0060026)
Supplement: Figure S1 — A photograph of liquid cultures of wild type (left) and bciD mutant (right) grown under phototrophic conditions. (DOC) [file pone.0060026.s001.doc]

**
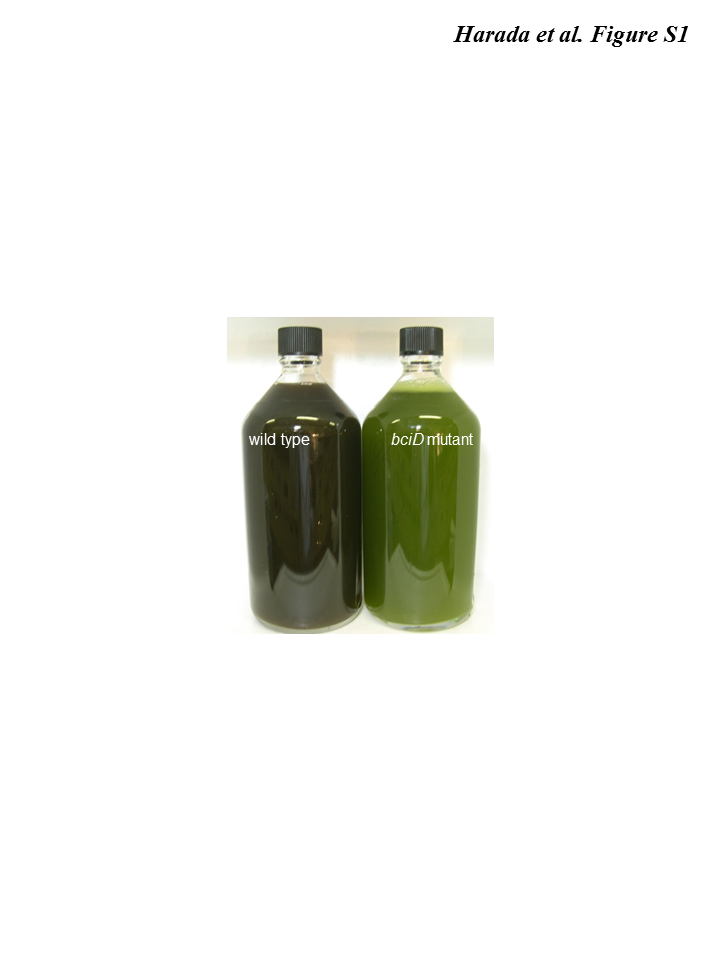
**

**Figure S1. A photograph of liquid cultures of wild type (left) and *bciD* mutant (right) grown under phototrophic conditions.**
