# Supplementary material for: Specific Gene bciD for C7-Methyl Oxidation in Bacteriochlorophyll e Biosynthesis of Brown-Colored Green Sulfur Bacteria
Source: PLoS One. 2013 Apr 1;8(4):e60026. doi: 10.1371/journal.pone.0060026 (PMC3613366; doi:10.1371/journal.pone.0060026)
Supplement: Figure S2 — Phylogenetic analysis of bciD paralogs among photosynthetic bacteria. A neighbor-joining tree was constructed with translated sequences of bciD paralogs that showed over 1e-10 of the BLASTP e-value. Bootstrap values for each clade were obtained by 1500 replications, and indicated. The accession numbers of sequences to construct the tree are as follows: Acaryochloris marina MBIC11017, YP_001516556; Acaryochloris sp. CCMEE 5410, ZP_09251378; Chl. phaeobacteroides BS1, YP_001958720; Chl. phaeobacteroides DSM266, YP_910687; Pelodictyon phaeoclathratiforme BU-1, YP_002019518; Prochlorococcus marinus MIT9301, YP_001091538; Rba. capsulatus SB 1003, YP_003578374; Rhodomicrobium vannielii ATCC17100, YP_004013831; Rba. sphaeroides ATCC17025, YP_001166986; Rps. palustris CGA009, NP_947956; Rps. palustris TIE-1, YP_001991868; Rps. palustris BisA53, YP_782106; Rubrivivax gelatinosus IL144, YP_005437386; Synechococcus elongatus PCC6301, YP_171412; Synechococcus elongatus PCC7942, YP_399856; Synechococcus sp. CC9605, YP_381421; Synechococcus sp. PCC7002, YP_001733926; Synechococcus sp. PCC7335, ZP_05036141; Synechococcus sp. WH5701, ZP_01084318; Synechococcus sp. WH 7805, ZP_01123862; Synechocystis sp. PCC6803, NP_442645. (DOC) [file pone.0060026.s002.doc]

**
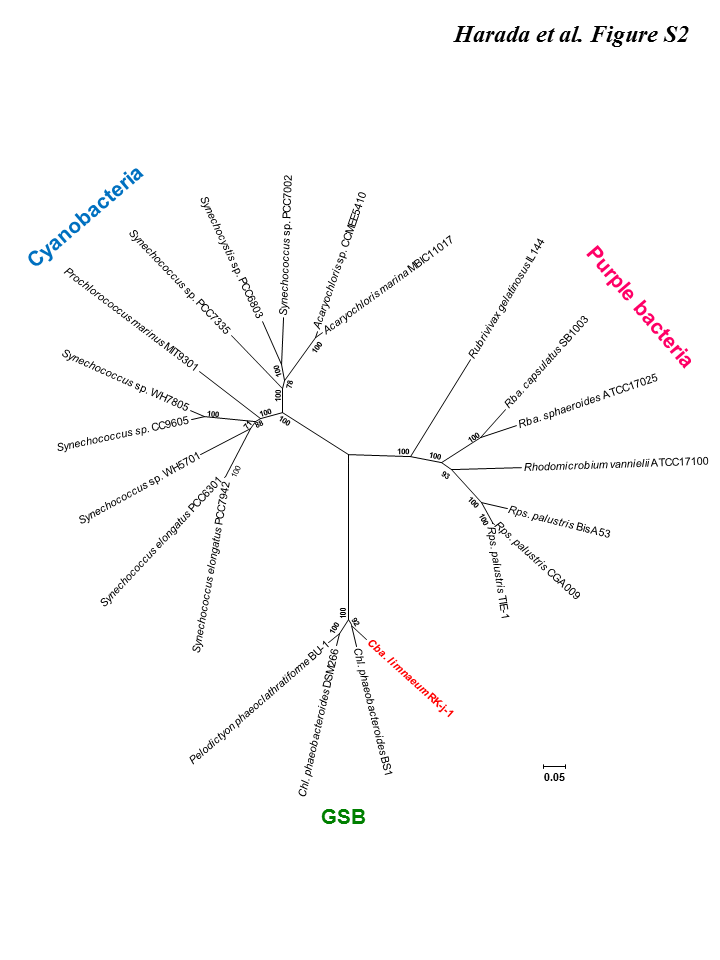
**

**Figure S2. Phylogenetic analysis of *bciD* paralogs among photosynthetic bacteria.** A neighbor-joining tree was constructed with translated sequences of *bciD* paralogs that showed over 1e-10 of the BLASTP e-value. Bootstrap values for each clade were obtained by 1500 replications, and indicated. The accession numbers of sequences to construct the tree are as follows: *Acaryochloris marina* MBIC11017, YP_001516556; *Acaryochloris* sp. CCMEE 5410, ZP_09251378; *Chl. phaeobacteroides* BS1, YP_001958720; *Chl. phaeobacteroides* DSM266, YP_910687; *Pelodictyon phaeoclathratiforme* BU-1, YP_002019518; *Prochlorococcus marinus* MIT9301, YP_001091538; *Rba. capsulatus* SB 1003, YP_003578374; *Rhodomicrobium vannielii* ATCC17100, YP_004013831; *Rba. sphaeroides* ATCC17025, YP_001166986; *Rps. palustris* CGA009, NP_947956; *Rps. palustris* TIE-1, YP_001991868; *Rps. palustris* BisA53, YP_782106; *Rubrivivax gelatinosus* IL144, YP_005437386; *Synechococcus elongatus* PCC6301, YP_171412; *Synechococcus elongatus* PCC7942, YP_399856; *Synechococcus* sp. CC9605, YP_381421; *Synechococcus* sp. PCC7002, YP_001733926; *Synechococcus* sp. PCC7335, ZP_05036141; *Synechococcus* sp. WH5701, ZP_01084318; *Synechococcus* sp. WH 7805, ZP_01123862; *Synechocystis* sp. PCC6803, NP_442645.
