# Supplementary material for: Specific Gene bciD for C7-Methyl Oxidation in Bacteriochlorophyll e Biosynthesis of Brown-Colored Green Sulfur Bacteria
Source: PLoS One. 2013 Apr 1;8(4):e60026. doi: 10.1371/journal.pone.0060026 (PMC3613366; doi:10.1371/journal.pone.0060026)
Supplement: Table S1 — GSB whose genome sequences were used for CCCT analysis. (DOC) [file pone.0060026.s003.doc]

**Table S1. GSB whose genome sequences were used for CCCT analysis.**

| **Query** | **BioProject ID** | **Genome size** |
| --- | --- | --- |
| *Chl. phaeobacteroides* BS1 (BChl *e*) | PRJNA12608 | 2.73 Mbp |
| **Target Species** |  |  |
| *Chl. phaeobacteroides* DSM266 (BChl *e*) | PRJNA12609 | 3.13 Mbp |
| *Pelodictyon phaeoclathratiforme* BU-1 (BChl *e*) | PRJNA13011 | 3.02 Mbp |
| *Cba. tepidum* TLS (BChl *c*) | PRJNA57897 | 2.15 Mbp |
| *Chl. limicola* DSM245 (BChl *c*) | PRJNA12606 | 2.76 Mbp |
| *Chl. phaeovibrioides*DSM265 (BChl *c* and *d*) | PRJNA12607 | 1.97 Mbp |
| *Prosthecochloris aestuarii* SK413 (BChl *c*) | PRJNA12749 | 2.51 Mbp |
| *Pelodictyon luteolum* DSM273 (BChl *c*) | PRJNA13012 | 2.36 Mbp |
| *Chloroflexus aurantiacus*J-10-fl*^a^* (BChl *c*) | PRJNA59 | 5.26 Mbp |

*^a^*Filamentous anoxygenic phototrophic bacterium.
