# Supplementary material for: Specific Gene bciD for C7-Methyl Oxidation in Bacteriochlorophyll e Biosynthesis of Brown-Colored Green Sulfur Bacteria
Source: PLoS One. 2013 Apr 1;8(4):e60026. doi: 10.1371/journal.pone.0060026 (PMC3613366; doi:10.1371/journal.pone.0060026)
Supplement: Table S2 — Top 5 genes specifically conserved among brown-colored GSB, that was calculated by CCCT. (DOC) [file pone.0060026.s004.doc]

**Table S2. Top 5 genes specifically conserved among brown-colored GSB, that was calculated by CCCT.**

| **Gene number*^a^*** | **Putative coding proteins** |
| --- | --- |
| Cphamn1_0270 | BciD involved in C7-formylation (this work) |
| Cphamn1_0271 | Uncharacterized protein |
| Cphamn1_0293 | Alkylhydroperoxidase (AhpD) core |
| Cphamn1_2460 | Rubrerythrin |
| Cphamn1_2461 | Glutamate synthase |

*^a^*Gene number of *Chl. phaeobacteroides* BS1 used as query in CCCT calculation.
