# Supplementary material for: Specific Gene bciD for C7-Methyl Oxidation in Bacteriochlorophyll e Biosynthesis of Brown-Colored Green Sulfur Bacteria
Source: PLoS One. 2013 Apr 1;8(4):e60026. doi: 10.1371/journal.pone.0060026 (PMC3613366; doi:10.1371/journal.pone.0060026)
Supplement: Table S3 — APCI-mass spectrometric data of BChl c and e homologs found in the full-growth cells of the wild type and bchU mutant of Cba. limnaeum . (DOC) [file pone.0060026.s005.doc]

***Table S3. APCI-mass spectrometric data of BChl c and e homologs found in the full-growth cells of the wild type and bchU mutant of Cba. limnaeum.^a^***

| HPLC  peak #*^b^* | Retention time (min) | Observed peaks | | | Calculated  for [M+H]^+^ | Pigment species |
| --- | --- | --- | --- | --- | --- | --- |
|  |  | Molecular ion*^c^* | Fragment 1*^d^* | Fragment 2*^e^* |  |  |
| 1 | n.d.  (16.2) | n.d.  (793.5) | n.d.  (775.5) | n.d.  (589.3) | 793.45 | R[E,M]BChl *c* |
| 2 | 20.7  (20.7) | 807.5  (807.5) | 789.5  (789.5) | 603.3  (603.3) | 807.46 | R[E,E]BChl *c* |
| 3 | 21.8  (21.7) | 807.5  (807.5) | 789.5  (n.d.) | 603.4  (603.4) | 807.46 | S[E,E]BChl *c* |
| 4 | 25.4  (25.4) | 821.5  (821.6) | 803.6  (803.5) | 617.4  (617.4) | 821.48 | R[P,E]BChl *c* |
| 5 | 26.8  (26.8) | 821.5  (821.6) | 803.6  (803.5) | 617.4  (617.4) | 821.48 | S[P,E]BChl *c* |
| 6 | 31.1  (31.1) | 835.5  (835.6) | 817.6  (817.5) | 631.8  (n.d.) | 835.50 | R[I,E]BChl *c* |
| 7 | 32.8  (32.9) | 835.5  (835.5) | 817.5  (817.6) | 631.4  (631.3) | 835.50 | S[I,E]BChl *c* |
| 8 | 16.4 | 821.5 | 803.5 | 617.3 | 821.44 | R[E,E]BChl *e* |
| 9 | 17.2 | 821.6 | n.d. | n.d. | 821.44 | S[E,E]BChl *e* |
| 10 | 19.8 | 835.5 | 817.5 | 631.3 | 835.46 | R[P,E]BChl *e* |
| 11 | 20.9 | 835.5 | 817.5 | 631.3 | 835.46 | S[P,E]BChl *e* |
| 12 | 23.9 | 849.6 | 831.5 | n.d. | 849.47 | R[I,E]BChl *e* |
| 13 | 25.2 | 849.5 | 831.5 | 645.3 | 849.47 | S[I,E]BChl *e* |

*^a^*Values of the authentic *Cba. tepidum* containing BChl *c* are shown in parentheses. n.d. = not detected.

*^b^*See Fig. 4.

*^c^*[M+H]^+^ .

*^d^*[M–OH]^+^ or [M–H_2_O+H]^+^ .

*^e^*[M–farnesyl+2H]^+^, protonated BChlide *c* or *e*.
